# Defining the Level of Need and Total Intervention Time in Children's Speech and Language Therapy in Finland: Developing a Consensus-Based Guideline

**DOI:** 10.1177/23969415261464496

**Published:** 2026-07-08

**Authors:** Sirpa Tarvainen, Eva Ståhlberg-Forsén, Hanna Elo, Marita Haukilehto, Guideline Work Group, Stina Sundstedt

**Affiliations:** 1 Department of Speech-Language Pathology, Faculty of Medicine, 60655 University of Helsinki, Helsinki, Finland; 2 Therapy Center Otavala, Kuokkamaantie 4a, Tampere, Finland; 3 Center for Intellectual Disability Care, 60664 Oulu University Hospital, Oulu, Finland; 4 Research Unit of Logopedics, Faculty of Humanities, University of Oulu, Oulu, Finland; 5 The Finnish Association of Speech and Language Therapists, Helsinki, Finland; 6 Faculty of Arts, Psychology and Theology, 162218 Åbo Akademi University, Turku, Finland

**Keywords:** Speech, language and communication needs, dosage, dose, speech-language pathology

## Abstract

**Background and Aims:**

Speech and language therapy is essential for supporting the skills and participation of children with disorders that impair their ability to interact, communicate, understand or use language, speak, eat or swallow. However, it might be challenging to outline how much intervention should be recommended for each individual. Currently, there are no guidelines to support clinical decision-making regarding the child's level of need for intervention, and thus, the recommended amount of speech and language therapy. The aim of the present work was to create a consensus-based guideline for clinical use in speech and language therapy in Finland to enhance equitable treatment and purposeful allocation of resources. This guideline was designed to assist in evaluating the child's level of need for intervention and, based on that, the total intervention time of speech and language therapy for children up to 17 years of age.

**Methods:**

A work group of Finnish speech and language therapists created a consensus-based guideline regarding the level of need for intervention and total intervention time during a 3-year process. During this time, the guideline was improved through a pilot phase following three comment rounds, including expert evaluations, conducted within the field of speech and language therapy as well as among other stakeholders. The ACCORD guideline was used to report this consensus work.

**Results:**

A guideline including a framework for outlining the level of need for intervention was developed to support clinical decision-making regarding the total intervention time per year of speech and language therapy for children up to 17 years. The guideline is based on the International Classification of Functioning, Disability and Health and the idea of adaptive intervention. Four factors were considered essential: (1) impact the disorder has on participation, (2) need for consultative support, (3) severity of the disorder, and (4) expected benefit of the intervention. Application of the guideline in the Finnish context and expert evaluations on the guideline and its validity are presented.

**Conclusions:**

We developed a guideline to define the child's level of need for intervention and total intervention time. The content validity of the guideline was high based on the ratings by the expert evaluators. We hope that this guideline will support clinical decision-making regarding total intervention time per year in Finland and support the creation of similar guidelines in other contexts.

**Implications:**

The present guideline may facilitate both intradisciplinary and interdisciplinary discussion regarding the child's level of need for intervention, and based on that, the recommended total intervention time. Systematic considerations of predefined factors used to define the level of need for intervention may enhance more equitable national practices.

## Introduction

Communication is a human right (McLeod, 2018). To ensure this right in children with speech, language and communication needs, speech and language therapy interventions are needed. The amount of intervention is known to affect intervention efficacy (Frizelle et al., 2021). It is, however, not simple to evaluate how much intervention each child needs, and thus, what amount of intervention should be recommended or offered (e.g., Kenny et al., 2009).

The decisions regarding the intervention amount are guided by the child's level of need for intervention. The level of need for intervention is dependent on the individual situation of each child. It can vary from low to very high, resulting in different levels of need for support, and thus, different amounts of intervention. Defining a child's level of need for intervention is, however, complex because several factors may affect the individual situation. The International Classification of Functioning, Disability and Health (ICF) is a commonly used model when considering individual situations (World Health Organization, 2001). Activities, participation, environmental and personal factors all affect a person's situation. Therefore, even though two individuals have the same disorder, their level of need for support can differ.

An example of how the individual situation affects the level of need for intervention is the impact a disorder has on participation. The impact varies as diagnosis or the severity of the disorder do not directly predict participation (Almqvist & Granlund, 2005). Two individuals with the same diagnosis and similar degree of severity may be able to participate on very different levels, and even a mild disorder can cause a moderate or severe impact on participation (World Health Organization, 2001). Therefore, how the disorder impacts participation in each individual should be evaluated individually.

The level of need for intervention can also differ based on environmental factors. This is exemplified in a situation where two 3-year-old children have similar language difficulties, but the parents of these children have different skills to support their child's language: one pair of parents are unaccustomed, whereas the other pair have received training to support their child's language skills. The need for consultative support in the child's surroundings thus also affects the level of need for intervention. Consultative support refers to the collaborative effort between speech and language therapists (SLTs) and individuals in the child's immediate environment to support the child's functional capacity (Roberts et al., 2019; Te Kaat-van den Os et al., 2017). Consultative support is crucial for the implementation of everyday rehabilitative routines.

Severity of the disorder also affects the level of need for intervention (Ebbels et al., 2019). For example, children with a greater severity of speech sound disorder require higher intervention intensity to effect change (Williams, 2012). Moreover, children with diverse disability typologies, such as Down syndrome, autism, or developmental language disorder, may need different amounts of intervention to achieve specific outcomes: children whose disorder can be categorized as more severe than others, such as Down syndrome, have been shown to need more intervention sessions and thus longer interventions to reach a predetermined level in learning new vocabulary (Davis et al., 2016).

The expected benefit of an intervention for the individual in question and people in his/her surroundings needs to be considered as well. A core premise in health care is that an intervention should only be offered when it is effective (Institute of Medicine (US) Committee on Quality of Health Care in America, 2001). If an intervention is not expected to lead to any improvement, there is no point in offering it. This is also aligned with the need for equitable distribution of public health care resources and principles of professional ethics (Institute of Medicine (US) Committee on Quality of Health Care in America, 2001; Kummer & Turner, 2011).

To summarize, the level of need for intervention is not dependent on any single factor, and thus considering a child's diagnosis or age alone is not enough to evaluate the level of need for intervention (compare to Kiviranta et al., 2016). Several factors need to be considered when assessing the individual situation and needs of each child.

After the child's level of need for intervention is determined, the child should be offered the appropriate amount of intervention. The amount of intervention is a seemingly simple concept, but on closer examination, it turns out to be quite complex. The amount of intervention can be approached in two different ways. The first approach is using the concept of dosage. Dosage includes (within session) dose, dose frequency (or session frequency), and total intervention duration resulting in cumulative intervention intensity (Frizelle et al., 2021; Warren et al., 2007). Dose is commonly defined as the number of properly administered teaching episodes during a single intervention session, including the following subcomponents: the average rate of teaching episodes per unit of time and the length of the intervention session. Cumulative intervention intensity is the product of dose × dose/session frequency × total intervention duration, resulting in a total number of administered teaching episodes over the total intervention duration (Frizelle et al., 2021; Warren et al., 2007). A 45-min session may include 90 teaching episodes, such as recasts. If there is one session per week for 10 weeks this results in a cumulative intervention intensity of 900 teaching episodes, which is in this example 900 recasts. Depending on study design and theoretical perspective, dose can be operationalized also as “time spent on language” expressed in minutes rather than in the number of teaching episodes, as done by Schmitt et al. (2017) and Justice et al. (2017). The present work uses the teaching episode-based operationalization of dosage described by Warren et al. (2007).

Another approach to the amount of intervention is using the concept of total intervention time. Total intervention time consists of the length of the intervention session, session frequency, and total intervention duration. It differs from dosage as it does not include the within-session dose (Table 1). Total intervention time can consist, for example, of 45-min sessions provided once a week for 10 weeks, resulting in a total intervention time of 10 × 45 min equaling 450 minutes or 7.5 hours.

**Table 1.** Examples of Cumulative Intervention Intensity and Total Intervention Time.

Concept	Length of a Session	Average Rate of Teaching Episodes Per Unit of Time	Session Frequency	Duration	Cumulative Intervention Intensity, i.e., Total Number of Teaching Episodes	Total Intervention Time
**Dosage**	45 min	90 episodes per 45 min	1 session per week	10 weeks	900 teaching episodes (90 episodes per 45 min × 1 session per week × 10 weeks)	-
**Total intervention time**	45 min	-	1 session per week	10 weeks	-	10×45 min = 450 min or 7.5 h

The concepts of cumulative intervention intensity and total intervention time are both needed. Cumulative intervention intensity defines the dosage, that is, the total number of teaching episodes, needed to make desired changes. Total intervention time, in contrast, describes the temporal frames within which the SLTs can provide the needed dosage (teaching episodes). The concept of dosage, regardless of whether it is operationalized as teaching episodes or time spent on language, includes aspects related to the content of the intervention sessions. As such, it is not suitable when professionals other than SLTs co-discuss or define the resources related to the recommended amount of intervention, as in the Finnish health care system. In this case, total intervention time is more applicable.

Based on the individual situation, interventions are adapted to include only those features or the amount of intervention that is necessary to meet the person's needs, and to use only the amount of resources necessary. This is the basic principle of adaptive interventions (Collins et al., 2004). In adaptive interventions, different dosages of certain treatment components are assigned to different individuals, and/or within individuals across time, with dosage varying in response to the needs of the individual. To determine how much intervention is needed, adaptive interventions use prespecified decision rules based on each participant's values on central characteristics (Collins et al., 2004). The idea of adaptive intervention is included in the practices of SLTs, as the individual situation always affects the way a person and the people in their immediate environment are supported. However, it is important to increase the understanding of the complex situation and strive to achieve greater transparency and consistency in practices (McGill et al., 2021). A systematic use of certain predefined criteria or guidelines to help determine the level of need for intervention could further support clinical decision-making regarding the required total intervention time per year, and result in more equitable national services for children.

There is currently a lack of research and clinical guidelines to help evaluate the child's level of need for intervention and, based on it, recommend the total intervention time. Dosage is a concept that has been studied more than total intervention time. Recent research on dosage is summarized in the systematic review of Frizelle et al. (2021). The dosage literature suggests for example, that if dose rates are compressed into half the time during treatment, performance does not suffer (Plante et al., 2019). Further, children receiving high frequency and low dose, or low frequency and high dose treatment have had better outcomes than children receiving high frequency, high dose or low frequency, low dose treatment (Schmitt et al., 2017). Also, an algorithm for optimizing the dosage and intervention time in language intervention has been presented (Justice et al., 2017). Most of the dosage literature, however, is not directly relevant to the question of total intervention time because the concept of dosage includes within-session dose or time spent on language.

The existing research literature on recommending total intervention time is very scarce, including one Finnish recommendation (Kiviranta et al., 2016) and a recommendation by the American Speech-Language-Hearing Association (ASHA), which was later withdrawn (Fernald, n.d.). Moreover, the suggested criteria in these documents do not take into account all the factors that may influence the required amount of intervention, such as the effect the disorder has on participation, the consultative support needed by people in the child's surrounding need, the severity of the disorder, or the expected benefit of an intervention.

To summarize, research on dosage is not directly applicable to support decision-making related to total intervention time. To date, there is very little research evidence that can aid clinical decision-making in assessing the level of need for intervention and recommending the total intervention time of speech and language therapy in children. Nevertheless, daily decisions on recommended intervention time in speech and language therapy need to be made. The lack of guidelines is likely to result in diverse practices and rationales for recommending intervention amounts, and consequently, inequity between children. Before further research evidence regarding the level of need for intervention and the total intervention time, clinical expertise and effective current practices should be integrated with existing research literature to inform clinical decision-making. Therefore, a consensus-base approach was chosen for the present work to develop a guideline that supports clinical decision-making in the Finnish context regarding the level of need for intervention, and based on that, the recommended total intervention time of speech and language therapy for children aged 17 and younger. With the implementation of the guideline, coherence in national practices could be improved, thereby strengthening equity among children in need of speech and language therapy.

## Aim

The aim of the present work was to create a consensus-based guideline for clinical use to evaluate the level of need for speech and language therapy in children aged 17 and younger, and to enhance coherence, transparency and equitable practices in Finnish speech and language therapy recommendations. Also, the aim was to support appropriate allocation of resources. The prime purpose of the guideline was to assist in evaluating the child's level of need for intervention, and based on that, the total intervention time per year.

## Methods

### Overview of the Consensus-Based Process and Validation of the Guideline

The present guideline was developed by a work group of 14 Finnish SLTs, recruited through an open call issued by the Finnish Association of Speech and Language Therapists. All members had clinical expertise and some additionally had a background in research. The guideline work proceeded from January 2019 to October 2022 (Table 2). A total of 29 meetings, including practical preparations by only a few persons, were held. In a majority of the meetings all the work group members were present. The first author acted as the chair of the group and was present at every meeting. In the consensus decision-making the following procedures were used: open panel discussions, open voting, and calculation of averages. Consensus was deemed to have been reached when no further discussion on a given topic was required. The ACCORD guideline (Gattrell et al., 2024) was used to report this consensus work. The present consensus exercise was not registered.

**Table 2.** Timeline for the Guideline Work.

Time	Phase	Objective
01/2019–05/2019	Preliminary planning	Formulate the preliminary project plan
02/2019–02/2019	Work group recruitment	Invite SLTs through an open call via the FASLT
06/2019–03/2020	Work phase I	Develop the first version of the guideline
04/2020–01/2021	Pilot phase	Test the guideline framework on case examples within the guideline work group
02/2021–05/2021	Comment round I	Collect expert evaluations from the FASLT board members
06/2021–08/2021	Work phase II	Further improve the guideline, create the cover letter and the feedback questionnaire
09/2021–10/2021	Comment round II	Collect expert evaluations from Finnish SLT's
11/2021–01/2022	Work phase III	Add specifications and information to the guideline based on the SLT comment round
02/2022–04/2022	Comment round III	Collect expert evaluations from various stakeholders
05/2022–09/2022	Work phase IV	Finalize work, prepare for publication
10/2022	Publication	Publish guideline on the FASLT website, host a webinar

*Note*. FASLT = Finnish Association of Speech and Language Therapists; SLT = Speech and Language Therapist.

Expert evaluations and input from SLTs and other stakeholders were collected in three separate comment rounds (See Table 2). Content validity (Almanasreh et al., 2019; Polit & Beck, 2006) of the guideline was assessed by asking expert evaluators to rate whether the guideline included all the relevant aspects to determine the level of need for speech and language therapy. Ratings were given on a 5-point Likert scale ranging from “not at all” to “includes all relevant”. For the calculation of the Content Validity Index, the number of evaluators assigning scores 4 or 5 was divided by the total number of experts. The use of a 5-point Likert scale, instead of the conventional 4-point scale, yields a more conservative estimate of the content validity. A detailed description of the guideline work and the content of each phase is included in Appendix 1, a presentation of the work group members is found in Appendix 2, and the ACCORD checklist in Appendix 3.

### Ethics

The present work was conducted according to the guidelines of the Finnish National Board on Research Integrity, TENK, on the ethical principles of research with human participants and ethical review in the human sciences in Finland (Finnish National Board on Research Integrity TENK, 2019). Preliminary work plan for the guideline and guideline members were approved by the board of Finnish Association of Speech and Language Therapists on March 29, 2019. Informed consent from respondents to use their data in this research was obtained and no sensitive personal data was collected. The expert evaluations from various stakeholders were collected anonymously, except for the feedback from the Finnish Association of Speech and Language Therapist board members and the voluntary feedback sent outside the anonymous form from various stakeholders.

## Results

### Guideline for Defining the Level of Need and Total Intervention Time in Children's Speech and Language Therapy

**Figure 1.**
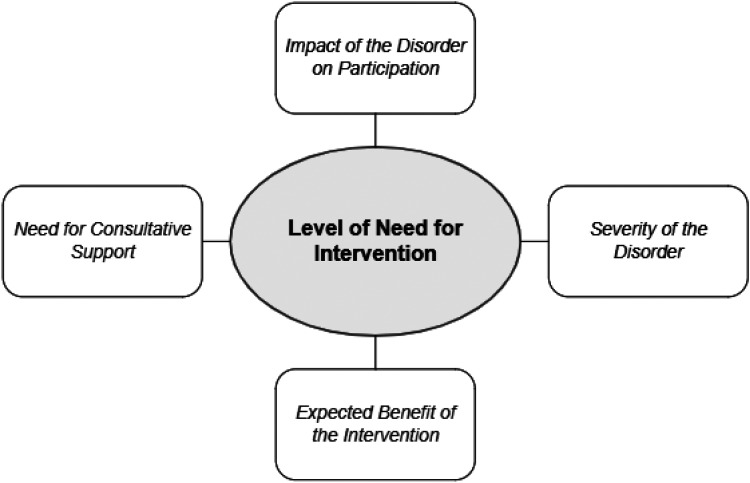
Four factors affecting the level of need for intervention.

The present guideline is founded on the ICF (World Health Organization, 2001) and on the concept of adaptive intervention, in which the amount of intervention is tailored to individual needs (Collins et al., 2004). This guideline is intended for children up to 17 years old with any disorder that impairs the individual's ability to interact, communicate, understand or use language, speak, eat, or swallow. Based on clinical experience and existing research literature, the guideline work group identified four factors that are essential when considering the level of need for intervention, and based on that, outlining the needed total intervention time per year (Figure 1)*.*

The work group used the ICF both as a conceptual model and as an instrument to evaluate activities and participation. When applicable, the official ICF severity labels (i.e., no, mild, moderate, severe, total) were applied to the four factors. When there were no existing ICF-labels that could be used to score the factor, the guideline work group developed new labels.

### Factor 1: Impact of the Disorder on Participation

This factor refers to the impact the disorder has on interaction with others, as well as on participation in activities and situations that are meaningful in one's life (Table 3). This evaluation should be done in collaboration with the child, people in the child's immediate environment (e.g., parents, teachers), and with professionals who have been delivering the intervention. The impact the disorder has on participation should be compared to the child's assumed best level of participation, considering their cognitive abilities.

**Table 3.** Impact of the Disorder on Participation.

Ability to Participate (in an Activity)^a^	Need for Support in Participation^a^	Impact of the Disability^b^	Impact of the Disorder on Participation^b^	Scoring
Always able to participate	Support needs in level with peers	0%–4%	No	0
Almost always able to participate	Occasional support required	5%–24%	Mild	2
Often able to participate	Regular support required	25%–49%	Moderate	4
Occasionally able to participate	Near-continuous support required	50%–95%	Severe	6
Almost never able to participate	Continuous support required	96%–100%	Total	8

a Scale developed by the guideline work group.

b Scale based on the International Classification of Functioning, Disability and Health (ICF) (World Health Organization, 2001).

### Factor 2: Need for Consultative Support

Consultative support refers to the context-focused collaborative work between a professional and people in the child's immediate environment (Table 4). The need for consultative support can be assessed through discussions, informal or formal observations (e.g., Barnett et al., 2022; Sylvestre et al., 2021) and background information. If alternative and augmentative communication is recommended, we suggest that there is a severe or absolute need for consultative support. If there is a discrepancy between the skills and means demonstrated by the individuals in the child's immediate environment (e.g., parents or teachers) and their perception of the need for collaboration, both should be considered to meet the needs of the child.

**Table 4.** Need for Consultative Support.

Skills and Means to Support the Child Demonstrated by Individuals in the Child's Immediate Environment^a^	Perception of the Need for Collaboration among Individuals in the Child's Immediate Environment^a^	Need for Consultative Support^b^	Scoring
Good skills and means	No need for collaboration	No	0
Relatively good skills and means	Small/low need for collaboration	Mild	1
Some skills and means	Clear/moderate need for collaboration	Moderate	2
Only a few skills and means	Great/high need for collaboration	Severe	3
Absolute need for skills and means	Significant need for collaboration	Total	4

a Scale developed by the guideline work group.

b Scale based on the International Classification of Functioning, Disability and Health (ICF) (World Health Organization, 2001).

### Factor 3: Severity of the Disorder

The severity of the disorder refers to any of the child's disorders that impair the individual's ability to interact, communicate, understand or use language, speak, eat, or swallow (Table 5). Even though children with a severe disorder often require more intervention to learn a new skill and continue developing, it is also important to acknowledge that intervention goals vary and that the greatest amount of intervention is not always recommended for the children with the greatest severity of the disorder. The severity of the disorder can be evaluated by using parental interviews, clinical observations, standardized test methods, established classification scales, multiprofessional evaluations, previous assessments, and evidence-based knowledge about the disorder. When assessing the severity of the disorder one should assess the child's functioning in relation to age-related expectations.

**Table 5.** Severity of the Disorder.

Functioning in Relation to Age-Related Expectations^a^	Magnitude of the Impairment^b^	Severity of the Disorder^b^	Scoring
At age level	0%–4%	No disorder	0
Slightly/mildly below age-related expectations	5%–24%	Mild	1
Well below/moderately below age-related expectations	25%–49%	Moderate	2
Significantly/very well below age-related expectations	50%–95%	Severe	3
Extremely limited in relation to age-related expectations	96%–100%	Total	4

a Scale developed by the guideline work group.

b Scale based on the ICF (World Health Organization, 2001).

### Factor 4: Expected Benefit of the Intervention

Expected benefit refers to the predicted impact of the intervention on the child and the individuals in the child's immediate environment (Table 6). The expected benefit of the intervention can be predicted based on research evidence regarding the rehabilitation potential of the disorder, clinical expertise, factors related to the child (i.e., profile of difficulties; child's readiness to collaborate; motivation), intervention (i.e., amount, content and effectivity of earlier intervention), and the environment (i.e., parents’ motivation; support from parents and others). When evaluating the expected benefit of an intervention, both short- and long-term effects should be considered. The evaluation should include potential benefits for the child's skills, participation, and daily activities, as well as for the skills, behaviors, and attitudes of individuals in the child's immediate environment. Interventions that target the child's environment through consultative support may improve the skills, behaviors, and attitudes of caregivers and professionals, thereby enhancing the child's opportunities for participation, even when the rehabilitation potential of the disorder itself is low or moderate. A child should not be left without intervention due to limited motivation of the people in the child's immediate environment. If there is a marked discrepancy between the rehabilitation potential and the motivation or readiness of the child or caregivers, the overall situation should be assessed to determine the expected benefit of intervention. If no benefit is expected, intervention should not be offered. When the expected benefit is uncertain, this factor should be scored as if the child or individuals in the child's immediate environment could benefit from the intervention.

**Table 6.** Expected Benefit of the Intervention.

Rehabilitation Potential for the Disorder or Targeted Skill	The Child's and the Carer's Motivation and Readiness to Collaborate	Expected Benefit	Scoring
No	No	No	0
Low	Low	Mild	2
Fair/moderate	Moderate	Moderate	4
High	High	High	6
Excellent	Excellent	Total	8

*Note*. Scales developed by the guideline work group.

### Level of Need for Intervention

**Figure 2.**
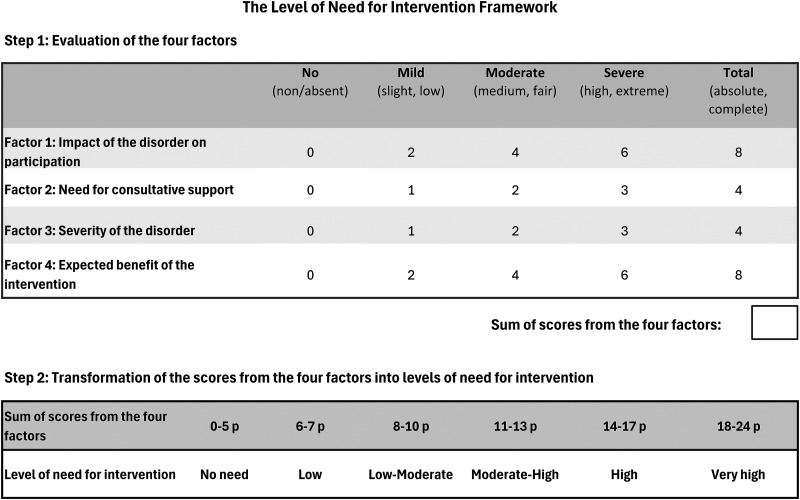
The level of need for intervention framework.

The four factors can be used to evaluate the level of need for intervention (Figure 2). Depending on the importance, the factors were scored on a 0–8 point scale (Factor 1 & Factor 4) or on a 0–4 point scale (Factor 2 & Factor 3). Consequently, the factors the work group considered more important have a greater impact on the level of need for intervention and, thus, on the recommended total intervention time per year. By adding up the scores of the four factors, the level of need for intervention (i.e., no, low, low-moderate, moderate-high, high, or very-high need) can be outlined. For example, a severe impact of the disorder (6 points), combined with a moderate need for consultative support (2 points), a moderate severity of the disorder (2 points), and a moderate expected benefit from an intervention (4 points) results in 14 points. This indicates a high level of need for intervention according to the framework.

A need for intervention is indicated when the sum of the four factors is six points or higher. However, if no benefit is expected from an intervention and the score on this specific factor is zero, intervention is not recommended even if the total sum reaches six points or more.

### Total Intervention Time

We propose using the level of need for intervention as the basis for determining the required total intervention time per year. Below, we demonstrate how the Level of Need for Intervention Framework can be used to outline national practices regarding total intervention time by presenting how the framework is suggested to be used in the Finnish context. It should be noted that the Finnish national recommendation presented below is not intended as a general recommendation on total intervention time, but as an example on how the level of need for intervention can be transformed into recommendations for total intervention time in a specific national setting.

### Recommendations for Total Intervention Time in the Finnish Context

#### The Finnish System Regarding Speech and Language Therapy

Currently in Finland, speech and language therapy for children is, in the vast majority, provided free of charge through the publicly funded healthcare system. The intervention is provided in two tiers. The initial assessment and intervention are provided in health care centers. This intervention typically includes around 10 sessions per year. The children assessed to have a significant negative impact of the disorder on participation are referred to a multidisciplinary team and may receive a recommendation from a physician for intensive medical rehabilitation (Ministry of Social Affairs and Health, 2022). This intervention is usually provided in 35–45 sessions per year, each session lasting 45–60 min (KELA/FPA, n.d.). The total number of sessions may also be somewhat lower or range up to 80 per year, depending on individual needs. The interventions are delivered by independent service providers in accordance with nationally defined standards of good rehabilitation practices based on the ICF framework including consultative support for caregivers and professionals.

#### Translating the Level of Need for Intervention Into Total Intervention Hours

**Figure 3.**
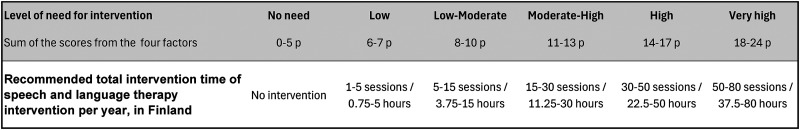
Transforming the level of need for intervention into total intervention time in the Finnish context.

The work group determined the recommended total intervention time ranges according to the current Finnish practices, acknowledging the variation in total intervention times across different regions and organizations. In Finland, speech and language therapy is often recommended as a specific number of 45–60 min intervention sessions per year. In this guideline, we have linked the level of need for intervention to recommended intervention sessions per year (Figure 3). The total intervention time can be calculated by multiplying the number of intervention sessions by the length of each session (e.g., 30 sessions × 45 min resulting in 1350 minutes or 22.5 hours).

Circumstances that may increase or decrease total intervention time or justify extended sessions (e.g., 60 min instead of 45 min), are presented in Appendix 4. The level of need for intervention should be evaluated every year. The assessment of the level of need for intervention should also be continued throughout the provided intervention and adjusted if necessary as the intervention progresses. For example, if the need for intervention diminishes, speech and language therapy may be ceased earlier than planned.

### Expert Evaluations of the Guideline in Finland

Three different expert evaluations of the guideline were conducted via three comment rounds, see also Table 2. The first round was qualitative and included comments from the FASLT board members. The second and third expert evaluations were qualitative as well as quantitative. The qualitative feedback mainly concerned the development of the guideline, whereas the quantitative expert evaluations focused on the acceptability, usability and content validity of the guideline, which were the main areas of interest and thus reported here.

#### Expert Evaluations by SLTs

The guideline was evaluated by 113 SLTs (comment round II), representing all of the five Finnish districts of collaborative areas for healthcare and social welfare. In Finland, there were 1952 working age SLTs (under 65 years) in the year 2021 (Valvira, 2024), making the proportion of Finnish SLTs answering the questionnaire approximately 6%. Of the 113 SLT evaluators (comment round II), 94% (*n* = 106) worked in a clinical setting either in evaluative work or as providers of intervention (Table 7). The majority of the respondents (*n* = 74; 65%) had more than 10 years of professional experience.

**Table 7.** Professional Roles of the Speech and Language Therapist Respondents, Comment Round II.

Professional Role	*N* = 113	%
Working in an intervention setting	62	54.9
Working in an evaluation setting	34	30.1
Working both on evaluation and intervention	10	8.8
Doctoral researcher in speech-language pathology	3	2.7
Faculty member in speech-language pathology	2	1.8
Counsellor for professionals	1	0.9
Supervizing personnel in a rehabilitation unit	1	0.9

The expert evaluations by the SLTs in comment round II (Table 8) indicated high acceptability as the mean of the overall grade for the guideline was above 4 and means of all the answers remained above 3 on a scale from 1 (weak/not at all) to 5 (excellent/very beneficial etc.). The content validity of the guideline in the expert evaluation in comment round II was 0.850, which is above the acceptable level of 0.78 (Polit & Beck, 2006).

**Table 8.** Evaluations of the Speech and Language Therapists (Comment Round II) and Various Stakeholders (Comment Round III).

Statement	SLTs			Various Stakeholders		
	Comment Round II			Comment Round III		
	*N* = 113			*N* = 36		
	*M*	*Md*	*SD*	*M*	*Md*	*SD*
Overall grade of the guideline	4.09	4	0.86	4.10	4	0.70
I recommend the use of the guideline in clinical work	4.16	5	1.14	4.10	4	1.10
The guideline includes all relevant aspects for determining the level of need for speech and language therapy intervention	4.17	4	0.86	4.30	4	0.70
The framework presented in the guideline is user-friendly	4.14	4	0.72	3.90	4	1.10
The presented framework is understandable	4.32	4	0.71	4.20	4	0.80
It is easy to use the guideline framework in everyday work	3.59	4	0.92	3.40	4	1.00
The framework is useful in evaluating the level of need for intervention	4.04	4	1.09	4.00	4	1.00
The framework is useful in evaluating total intervention time	3.99	4	1.16	4.00	4	1.10
It is possible to provide the recommended total intervention time	3.12	3	1.20	3.20	3	1.10

*Note*. The scale for the statements ranged from 1 to 5 so that 1 represented the weakest score (weak; not at all) and 5 represented the best score. The labels for 5 were excellent, fully agree, includes all relevant, very user friendly, very understandable, very easy, very beneficial, very useful and very likely. SLTs = Speech and Language Therapists; *M* = mean; *Md* = median; *SD* = standard deviation.

#### Expert Evaluations by Various Stakeholders

The guideline was also evaluated by 38 stakeholders (comment round III), from which two evaluations were excluded as the evaluators didn’t give permission to use their evaluation in research. In addition to the expert evaluations and comments in the online questionnaire, qualitative feedback was received from several institutions and organizations: the Ministry of Social Affairs and Health, the Social Insurance Institution of Finland, the work group that created the clinical recommendation for the treatment of DLD in Finland, children's rehabilitation unit in a university hospital, phoniatrics unit in a university hospital, and child neurology team in a wellbeing services county. Individual SLTs also provided qualitative feedback on the guideline. Of the various stakeholders (comment round III), a majority of the evaluators (69%; *n* = 25) were SLTs (Table 9). The evaluators were highly experienced professionals, with 67% (*n* = 23) reporting more than 10 years of professional experience.

**Table 9.** Professions of the Various Stakeholder Respondents, Comment Round III.

Profession of Respondents	*N* = 36	%
Speech and language therapist	25	69.4
Medical doctor	6	16.7
Representatives of patient organizations	3	8.3
Public health nurse	1	2.0
Interpreter for speech-impaired	1	2.8

The expert evaluations by the various stakeholders in comment round III (Table 8) indicated high acceptability as the mean of the overall grade for the guideline was above 4 and means of all the answers remained above 3 on a scale from 1 (weak/not at all) to 5 (excellent/very beneficial etc.). The content validity of the guideline on comment round III was 0.889, which is above the acceptable level of 0.78 (Polit & Beck, 2006). The guideline was thus considered valid in determining the child's level of need for speech and language therapy intervention.

## Discussion

The aim of the present work was to develop a consensus-based guideline for clinical use in Finland to enhance equitable practices and purposeful allocation of resources. This guideline was designed to assist in evaluating the child's level of need for intervention and based on that, the total intervention time in speech and language therapy for children up to 17 years of age. Based on clinical expertise and research literature, four factors were identified as essential in evaluating the child's level of need for intervention and, thus, the total intervention time: (1) impact the disorder has on participation, (2) need for consultative support, (3) severity of the disorder, and (4) expected benefit of the intervention. According to the expert evaluations, the guideline received wide approval for its content from SLTs and other stakeholders in Finland. The guideline was also found valid in assessing the child's level of need for speech and language therapy intervention.

### Level of Need for Intervention

Several of the factors identified in the present guideline as essential in assessing the level of need for intervention, such as severity of the disorder, child's communication environment and expectations of progress, have also been discussed as important intervention elements in previous studies (Ebbels et al., 2019; McCartney, 2000; McGill et al., 2021). However, there are no prior guidelines specifically addressing how to assess a child's level of need for intervention. As a result, the assessment relies primarily on the clinician's implicit tacit knowledge, which (optimally) integrates research evidence with clinical expertise (Thornton, 2006). Clinical expertise involves iterative cycles of inductive and deductive reasoning, which makes it susceptible to underlying personal biases (Saccone et al., 2025). In addition, individual, unit-level, and regional practices also play a role in the process. While practical and functional, these varying practices likely lead to inequity among children living in different regions and create a barrier to the implementation of evidence-based practice (Morgan et al., 2019).

In this guideline, we present the concept of “a child's level of need for intervention” to set the premise for evaluating the child's needs, and based on it, the total intervention time. The child's level of need for intervention is defined by comparing the child's situation to a set of predefined factors as suggested by Collins et al. (2004) considering the child's overall situation (World Health Organization, 2001): the impact of the disorder on participation, the need for consultative support, the severity of the disorder, and the expected benefit of the intervention. Systematically considering these predefined factors regarding each individual helps in assessing the child's level of need for intervention, and may result in more unified and less biased views on how to determine the needed total intervention time.

### Amount of Intervention

The present work distinguishes between the concepts of total intervention time and dosage, both of which are needed for creating an effect: total intervention time as the frame in which the SLTs can operate, and dosage as the actual dose affecting change (Warren et al., 2007). The research on dosage is still in its infancy (Frizelle et al., 2021) and the concept of total intervention time has received even less attention than dosage. The distinction between total intervention time and dosage (i.e., the number of properly administered teaching episodes) is crucial, as total intervention time is often determined by professionals other than SLTs, while dosage is determined by the SLT delivering the intervention. The effectiveness of an intervention is likely more reliant on dosage than total intervention time; more intensive language treatment measured as time is not always significantly associated with improved outcomes (Schmitt et al., 2017). This enables SLTs to have an important lever of effectiveness within their control even if they can’t control total intervention time. Nonetheless, to provide the necessary dosage, intervention hours are required. In this work, the focus has been on total intervention time to facilitate the discussion on the frames within which SLTs can provide the interventions and the necessary dosage.

Well-defined criteria regarding the total intervention time set the premise for equitable distribution of resources, also acknowledging individual situations. While previous research is based on the child's age or diagnosis (Kiviranta et al., 2016) or the severity of the disorder (Fernald, n.d.) in defining total intervention time, the present guideline suggests that the child's level of need for intervention should serve as a basis for the recommended total intervention time. National adaptations of how the level of need for intervention is translated into total intervention time are necessary, as the relationship between the level of need for intervention and total intervention time may vary considerably across settings due to differences in healthcare policies and resources in different countries or regions. The adaptations regarding total intervention time should, anyhow, consider the evidence from intervention studies, including findings regarding the existence of a critical minimum to enable effect (Frizelle et al., 2021). The smallest number of hours that have provided positive effects for morphosyntax or microstructure components of narratives in the review of Frizelle et al. (2021) was around 8 hours (Balthazar & Scott, 2018; Bellon-Harn, 2012; Bellon-Harn et al., 2014; Smith-Lock et al., 2013), though for the learning of specific vocabulary, even a shorter intervention time may be adequate. The number of hours in the Frizelle et al. (2021) review is, however, not directly comparable to the real-life situations of children receiving speech and language therapy, as the children often have difficulties in several domains (e.g., morphosyntax, vocabulary, narratives, pragmatics), or in several areas within each domain, and thus have several skills to learn and to maintain. Further, sufficient intervention time is required to support generalization of the acquired skills into everyday life situations in order to enhance participation. Thus, though research studies often consider progress on a single target, making progress on only one target is not sufficient in clinical setting, as children may need work on several targets each year and may need work on other targets in subsequent years.

### Expert Evaluations on the Guideline From the Finnish Stakeholders

The response to the guideline was in general good: the overall grade was high, the expert evaluators recommended the use of the guideline, agreed to a great extent that the guideline included all relevant aspects, and considered it relatively user-friendly and understandable. Also, the content validity was high both on comment rounds II and III. The weakest grades (3.12; 3.20) on comment rounds II and III were given on the statement “It is possible to provide the recommended amounts of SLT intervention.” The reason for this may be that in Finland, speech and language therapy resources and practices vary by regions and organizations. Providing recommended intervention hours can be challenging in areas with a shortage of resources in relation to the number of children needing support or in areas facing a shortage of SLTs. Therefore, resources and service policies often affect decisions making them “the fourth pillar” in evidence-based practice next to research evidence, clinical expertise and client perspectives (McCurtin & Clifford, 2015), even though they may act against research evidence. In this guideline, it was determined that the actual level of need for intervention should be documented regardless of the available resources.

The proportion of respondents in the second comment round (approximately 6% of SLTs in Finland) can be considered broadly representative of the SLT professional group. Further, some of the answers were known to be given as a group of SLTs thus raising the actual number of respondents higher than the received answers. The number of expert evaluations on the third comment round was small, but the significance of the feedback was enhanced by the qualitative answers received from institutions that can be considered most central regarding intervention in Finland: the Social Insurance Institute of Finland (which finances intensive medical rehabilitation, including speech and language therapy) and the Ministry of Social Affairs and Health. Further, the acceptability of the guideline in the Finnish context is emphasized by the guideline being referenced in a national recommendation for the criteria for referring people to medical rehabilitation by the Ministry of Social Affairs and Health (Ministry of Social Affairs and Health, 2022).

### Strengths and Limitations

#### The Guideline

The key strengths of this consensus-based guideline are that the guideline and the Level of Need for Intervention Framework are grounded in the ICF model (World Health Organization, 2001) and in the concept of adaptive intervention (Collins et al., 2004). We argue, in line with the ICF model, that a young child's functioning in daily life needs to be considered in relation to the environment of the child. A young child or a child with developmental disorders is often very dependent on the skills and attitudes of their environment. The Level of Need for Intervention framework acknowledges this interplay between the child and the environment. In the guideline and in the framework, the child's level of need for intervention is outlined by evaluating key factors in a structured way to support clinical decision-making regarding the total intervention time.

The present guideline adds to the scarce literature on evaluating the level of need for speech and language therapy intervention, and based on it, the total intervention time. Compared to the few resources on the matter (Fernald, n.d.; Kiviranta et al., 2016), this consensus-based guideline looks beyond the diagnosis, severity of the disorder and the child's age by acknowledging the child's situation as a whole. The significance of collaboration with the people in the child's close environment is acknowledged by recognizing them as the best experts of the child's life—along with the child itself. The predefined factors and the relation with which these factors are translated into total intervention time were selected and determined by the work group based on current clinical practices, known research literature and clinical expertise. These features were ratified by three comment rounds asking expert evaluations from various stakeholders and were found to have high content validity.

This guideline is not intended to replace individual considerations or clinical judgement as this type of guideline can not account for all the possible factors affecting intervention. Professionals also need to make practical decisions on dose, how to distribute the therapy sessions over time (Frizelle et al., 2021), and how to adapt the intervention to meet the individual needs of a family and the environment. When using the guideline, the clinician's evaluations can still be influenced by personal biases, for instance when predicting the expected benefit of the intervention. Still, by providing clear instructions, the guideline aims to increase equitable practices and transparency in decision-making. Furthermore, the relationship between the level of need for intervention and the total intervention time also needs to be adjusted in relation to national recommendations. Thus, this guideline offers suggestions for clinical decision-making and quantitative recommendations but cannot be used rigidly. Further, as the (long-term) effects of this guideline are not yet investigated, users should remain cautious and critical. The properties and clinical benefits of the guideline need to be verified in future studies and the reliability of the guideline further examined.

#### The Cconsensus Work Process

A consensus process was considered reasonable, as the creation of a guideline for clinical work requires expert problem-solving involving various stakeholders. There are different ways of conducting consensus-based work, such as the Delphi method (e.g., Okoli & Pawlowski, 2004). In the present guideline, the consensus work was conducted as a group that worked together during the whole process. Like in the Delphi method (Okoli & Pawlowski, 2004), comment rounds were conducted with varied aims and targeted audiences, collecting both qualitative and quantitative evaluations.

A strength of the consensus process is the approximate alignment of the work with the principles of the ACCORD methodology (Gattrell et al., 2024). Further strengths include the SLTs in the work group representing different professional contexts and regions, format of the work enabling deep discussions, piloting the guideline within the work group and collecting both quantitative and qualitative feedback from various expert evaluators. The work group consisted only of SLTs, but inviting organizations representing speech and language therapy service users and various professional stakeholders as evaluators aimed to compensate for this fact.

Some limitations of the process in relation to the ACCORD protocol (Gattrell et al., 2024) can also be identified, such as not conducting a systematic literature search prior to the process and the lack of preregistration, steering group and anonymous interaction in the consensus work. The possibility for work group members to provide anonymous answers could have increased the objectivity during the process, though in general, the current process proved flexible and served the purpose well. In future consensus processes, also other methods and techniques, such as the Delphi method (Okoli & Pawlowski, 2004), could be considered to assess and ensure consensus.

### Clinical Implications

There is currently a lack of research and published guidelines for determining the level of need for intervention or the total intervention time for children. This guideline provides an easily administered tool with the potential to support clinical decision-making, coherence and transparency in speech and language therapy practices. This guideline can contribute to the discussion on the foundations for recommendations on total intervention time. A careful assessment and documentation of the four predefined factors may also guide the SLTs in determining what proportion of the intervention should focus on specific skills, participation, and consultative support. When using the guideline, the rationale underlying the scoring of each factor should therefore be stated explicitly. For example, “The impact of the disorder on participation is *severe* as the child cannot communicate with peers in free play” or “The need for consultative support is *high* as the caregivers and staff need training in the use of augmentative and alternative communication methods.” These rationales help clarify the factors contributing to the child's level of need for intervention and may thereby inform decision-making regarding intervention goals and methods, as well as the role and form of the consultative support required.

Further, the use of the guideline may strengthen equity among the vulnerable group of children who need speech and language therapy services. The guideline may also assist in prioritizing services. Thus, the ethical stress healthcare professionals experience in decision-making on distributing resources (Kenny et al., 2009) may be decreased by the use of this, or a similar, guideline.

While the guideline was specifically developed for speech and language therapy interventions in children, it has the potential to benefit also other client groups and clinical disciplines. The guideline may also provide a tool across health care systems for specific purposes, such as identifying individuals with the highest level of need for intervention. However, as decision-making practices on interventions may differ in different countries and settings (McGill et al., 2021), the guideline needs to be culturally adapted and modified. This guideline has the potential to provide justifications for decision-making, enhance equitable provision of services, and increase intraprofessional and interprofessional discussions.

### Future Research

Further research on the properties of the present guideline, such as the ability to reliably assess the level of need for intervention and thus total intervention time, is needed. Also, it is recommended to continue collecting feedback on the use and implementation of the guideline and assess the need for updates. In future research, a systematic follow-up period with a baseline of recommendations before and after implementing the guideline could provide valuable information on the actual implementation and the possible effects. Furthermore, it is crucial to further investigate the guideline as a tool to evaluate the level of need for speech and language therapy intervention in children with different disorders and adapt the guideline across different health care systems and contexts. We invite fellow researchers and clinicians to test and further develop this guideline.

### Adapting the Guideline Across Contexts

As the guideline was developed within a Finnish context where the ICF model (World Health Organization, 2001), emphasizing participation and the impact of the environment, is strongly embedded in the rehabilitation system, adaptations are required in contexts characterized by pull-out service delivery as well as in settings where services primarily rely on indirect interventions and limited direct therapy. We suggest that the factors underlying the Level of Need for Intervention Framework can be used together as a conceptual model, while acknowledging that the relationship between the factors may vary depending on the cultural context. In the present guideline, the factors are combined using a summative approach, chosen to maintain simplicity and feasibility in clinical application; however, depending on context and purpose, future work may also explore alternative ways of modeling the relationships between factors (e.g., through adjusted weighting of scores or using multiplication rather than summation for some factors).

In some contexts, the expected benefit of the intervention may need to be separated from the other factors, as it can be argued that expected benefit does not define the child's level of need for intervention. For example, in research contexts requiring a clear distinction between a child's need related to the disorder and the expected benefit of intervention, the first three factors may be analyzed independently of the expected benefit rather than combined with it. In the current model, the factors are considered and stated separately but summarized to obtain a level of need for intervention leading to recommended total intervention time per year.

We recognize that the translation of the level of need for intervention into total intervention time is inherently linked to the length of individual intervention sessions. In the Finnish context, standard practice involves 45-minute sessions, and the guideline provides recommendations for situations that justify longer 60-minute sessions. However, the translation from the level of need for intervention to total intervention time will require intentional calibration for different contexts. Future adaptations of the guideline should also consider situations in which shorter sessions are clinically appropriate (e.g., for children with reduced attention capacity).

We invite work groups from other countries to use our Levels of Need for Intervention framework and the Finnish guideline as a starting point for developing national guidelines for interventions. Future guideline working groups should consider who will use the guideline, the professional field in which it will be applied, whether it is intended for national or regional use, and the intervention settings in which it will be implemented. They should also determine whether the aim is to identify the level of need for intervention to support prioritization, or to additionally estimate the total intervention time required. We welcome critical scrutiny of the framework and anticipate that the relationship between the factors, as well as the translation from levels of need for intervention to total intervention time, may be further refined to better suit specific national or local contexts.

## Conclusions

A work groupA work group of Finnish SLTs, representing both clinical and research expertise, developed a consensus-based guideline to support clinical decision-making in children's speech and language therapy to enhance equitable practices and purposeful allocation of resources. The guideline includes a novel Level of Need for Intervention Framework, and based on it, support for clinical decision-making regarding the total intervention time. This consensus-based guideline was developed based on the ICF (World Health Organization, 2001) and the idea of adaptive intervention (Collins et al., 2004). Based on clinical expertise and research literature, four key factors were identified to support decision-making: (1) impact the disorder has on participation, (2) need for consultative support, (3) severity of the disorder, and (4) expected benefit of the intervention. The guideline and its framework may enhance intra- and interdisciplinary discussion on the child's level of need for intervention and total intervention time. Also, the guideline may increase the equity in speech and language therapy intervention practices and enhance the development of coherent national recommendations. Moreover, the present work may enhance discussion on the basis of the recommended total intervention time and support the creation of similar guidelines in other contexts.

## Supplemental Material

sj-docx-1-dli-10.1177_23969415261464496 - Supplemental material for Defining the Level of Need and Total Intervention Time in Children's Speech and Language Therapy in Finland: Developing a Consensus-Based Guideline Supplemental material, sj-docx-1-dli-10.1177_23969415261464496 for Defining the Level of Need and Total Intervention Time in Children's Speech and Language Therapy in Finland: Developing a Consensus-Based Guideline by Sirpa Tarvainen, Eva Ståhlberg-Forsén, Hanna Elo, Marita Haukilehto, and Stina Sundstedt in Autism & Developmental Language Impairments

sj-xlsx-2-dli-10.1177_23969415261464496 - Supplemental material for Defining the Level of Need and Total Intervention Time in Children's Speech and Language Therapy in Finland: Developing a Consensus-Based Guideline Supplemental material, sj-xlsx-2-dli-10.1177_23969415261464496 for Defining the Level of Need and Total Intervention Time in Children's Speech and Language Therapy in Finland: Developing a Consensus-Based Guideline by Sirpa Tarvainen, Eva Ståhlberg-Forsén, Hanna Elo, Marita Haukilehto, and Stina Sundstedt in Autism & Developmental Language Impairments

sj-docx-3-dli-10.1177_23969415261464496 - Supplemental material for Defining the Level of Need and Total Intervention Time in Children's Speech and Language Therapy in Finland: Developing a Consensus-Based Guideline Supplemental material, sj-docx-3-dli-10.1177_23969415261464496 for Defining the Level of Need and Total Intervention Time in Children's Speech and Language Therapy in Finland: Developing a Consensus-Based Guideline by Sirpa Tarvainen, Eva Ståhlberg-Forsén, Hanna Elo, Marita Haukilehto, and Stina Sundstedt in Autism & Developmental Language Impairments

sj-docx-4-dli-10.1177_23969415261464496 - Supplemental material for Defining the Level of Need and Total Intervention Time in Children's Speech and Language Therapy in Finland: Developing a Consensus-Based Guideline Supplemental material, sj-docx-4-dli-10.1177_23969415261464496 for Defining the Level of Need and Total Intervention Time in Children's Speech and Language Therapy in Finland: Developing a Consensus-Based Guideline by Sirpa Tarvainen, Eva Ståhlberg-Forsén, Hanna Elo, Marita Haukilehto, and Stina Sundstedt in Autism & Developmental Language Impairments
