# Supplementary material for: Defining the Level of Need and Total Intervention Time in Children's Speech and Language Therapy in Finland: Developing a Consensus-Based Guideline
Source: Autism Dev Lang Impair. 2026 Jul 8;11:23969415261464496. doi: 10.1177/23969415261464496 (PMC13346750; doi:10.1177/23969415261464496)
Supplement: sj-docx-1-dli-10.1177_23969415261464496 - Supplemental material for Defining the Level of Need and Total Intervention Time in Children's Speech and Language Therapy in Finland: Developing a Consensus-Based Guideline [file sj-docx-1-dli-10.1177_23969415261464496.docx]

**Appendix 1**

**Description of the guideline work**

**Preliminary Planning Phase**

The idea and the initiative to address the need for the guideline came from the first author, who was at that time the chair of the Finnish Association of Speech and Language Therapists (later FASLT). A small group consisting of the first author, a member of the FASLT organisation, two group members and a counselling member developed a preliminary project plan for the guideline. Consensus-based approach was chosen as the very scarce existing research literature on total intervention time was not considered sufficient to form the basis for recommending total intervention time of speech and language therapy in the Finnish healthcare system. Further, a review of dosage would not have fulfilled the need for a guideline for clinical work regarding total intervention time. The present guideline is based on the clinical experience of the work group members, known research evidence and current national intervention practices in Finland.

The work plan was presented to the board of the FASLT who accepted the research plan, agreed to pay meeting fees for the work group members and, after the contract of the first author as the chair of the FASLT ended, financed some writing time for her as the compiling author to finalise the guideline. The FASLT provided feedback on the guideline on comment round I and accepted the final version to be added to the FASLT website, but the work group created the guideline independently.

**Recruiting the members of the Guideline Work Group**

A call was opened for all FASLT members to participate in creating the guideline. The invitation was sent via a FASLT membership email. A majority of the approximately 2100 registered SLTs in Finland in year 2019 (Valvira, 2024) were members of the FASLT ( n=1247, Finnish Association of Speech and Language Therapists, personal communication, 11 June 2025). Thus, the call was able to reach potential interested SLTs throughout the country. All the interested FASLT members (n=14) were included in the guideline work group. The members were experienced SLTs working both in public and private sectors representing all of the five Finnish collaborative areas for healthcare and social welfare. Members had clinical and some also research expertise. All of the professionals participating in the guideline work group had worked in positions focusing on intervention and/or evaluation. Two of the members represented the Finnish Swedish speaking minority community. The work group used primarily Finnish as the working language, but when needed, also Swedish and English. The work was conducted mostly via teleconference platform enabling the participation also from rural areas of Finland.

**Work Phase I**

The work group was informed about the scarcity of the literature on total intervention time and presented the rationale for choosing a consensus-based approach. We acknowledge that no systematic literature search was conducted and therefore the information provided to the work group on relevant literature may have been incomplete. Willingness of the work group members to act as the chair was requested. As no one else wished to act as the chair of the group, the first author continued to steer the work group. The work group implicitly formulated the theoretical background, selected the ICF as the basis of the guideline work and developed the first version of the guideline and the level of need for intervention framework with its scoring system through consensus decision making (open panel discussions, open voting, counting for average). A consensus was considered to be reached when there no longer was a need for a discussion regarding a certain topic. The key factors for the guideline and the framework were accepted unanimously. The work group members presented both evaluation and intervention settings. While the SLTs working mainly in evaluation in public organisations financing intervention may opt for more limited total intervention time as their resources are limited, SLTs working mainly in intervention may favour more generous intervention time to be able to support individuals and their families better. This conflict of interest within the group was managed by discussions and by reaching consensus between the various views. There were no other conflicts of interest during the guideline development. After the guideline was published, three of the work group members have given two webinars upon request for interested public organisations for a fee.

**Pilot Phase**

All participating work group members anonymised and/or formulated two case descriptions of SLT clients to be used as case examples to test the scoring in the level of need for intervention framework. The guideline work group members first individually scored the case examples using the preliminary scoring system. After individual scoring, the scores and the cases were discussed and analysed within the guideline work group. Because the scoring was not unanimous, the work group worked towards a consensus by calculating mean scores, voting, and with majority decisions. After scoring, the guideline work group discussed the observations from this pilot phase and formulated a consensus about the changes needed to improve the framework scoring system.

**Comment Round I - Board of the Finnish Association of Speech and Language Therapists**

FASLT board members (n=15) were asked to provide feedback on the guideline. The first author discussed the guideline with the board and also gained some written feedback.

**Work Phase II**

Minor modifications to the guideline were made and the cover letter along with the questionnaire for gathering feedback from the comment round II were created.

**Comment Round II - Speech and Language Therapists in Finland**

The comment round II was open to all SLTs and SLT students in Finland. Expert evaluations were collected through an anonymous online questionnaire. The invitations to comment on the guideline were sent via FASLT membership letters; personal emails to professors, university lecturers, and student organisations in all the five departments of speech-language pathology in the Finnish universities; email to the organisation of the Swedish-speaking SLTs in Finland; email to the board members of the FASLT professional ethics committee; and via three Finnish SLT social media platforms. No reimbursements were offered for participants’ time for providing feedback. Invitations for providing feedback were reiterated.

**Work Phase III**

The first author classified the answers according to different themes and presented the results for the guideline work group. The guideline, including the level of need for intervention framework, was further modified. Additional tables for each of the four factors were created to clarify and ease the use of the guideline. Specifications and explanations were added to the text.

**Comment Round III - Various stakeholders**

The call for expert evaluations in the third round was published in the public pages and social media platforms of the FASLT. The call was open for all interested stakeholders. In addition, there was a broad direct invitation for various patient and multiprofessional stakeholders for the third comment round, including academic faculty members and students of all the five departments of speech-language pathology in the Finnish universities, board members of the FASLT professional ethics committee, pediatricians and phoniatricians from phoniatrics and child neurology departments in the university hospitals in Finland, the Finnish Ministry of Social Affairs and Health, the Finnish Social Insurance Institution and several Finnish medical and patient associations. Comments were collected through an anonymous online questionnaire. No reimbursements were offered for participants’ time for providing feedback.

**Work Phase IV**

The first author presented the results on the comment round III for the guideline work group. The guideline work group revised and finalised the guideline. The manuscript was stylised for publication and planning for the publication webinar on the guideline was conducted.

**Publication**

The guideline was made freely available via public pages of the FASLT. This was done in November 2022 in Finnish, and in May 2023 in Swedish. The recommendation was presented to the Finnish SLTs via a free webinar in December 2022. The guideline presented on the FASLT website underwent a minor update in 2024 following two meetings.

References:

Finnish Association of Speech and Language Therapists. (2025, June 11). *Number of the member of the Finnish Association of Speech and Language Therapists* [Personal communication].

Valvira. (2024). *Sosiaali- ja terveydenhuollon ammattihenkilöt* [Dataset]. Avoindata.fi. https://www.avoindata.fi/data/sv/dataset/sosiaali-ja-terveydenhuollon-ammattihenkilot
