# Supplementary material for: Defining the Level of Need and Total Intervention Time in Children's Speech and Language Therapy in Finland: Developing a Consensus-Based Guideline
Source: Autism Dev Lang Impair. 2026 Jul 8;11:23969415261464496. doi: 10.1177/23969415261464496 (PMC13346750; doi:10.1177/23969415261464496)
Supplement: sj-docx-4-dli-10.1177_23969415261464496 - Supplemental material for Defining the Level of Need and Total Intervention Time in Children's Speech and Language Therapy in Finland: Developing a Consensus-Based Guideline [file sj-docx-4-dli-10.1177_23969415261464496.docx]

**Appendix 4**

**Additional considerations that may affect the recommended total intervention time or the length of a session**

**Circumstances that may reduce the recommended total intervention time**

o The expected benefits are low or limited.

o Despite an appropriate intervention plan that has been implemented as intended, speech and language therapy has yielded only limited benefits.

o The impact caused by the disorder can be mitigated with a relatively small amount of therapy (e.g., occasional stuttering or articulation errors).

o The child has other ongoing interventions.

o Given the family's current circumstances and resources, speech and language therapy is not considered a primary focus at this time. For example, if the family cannot currently engage in consultative support, the total recommended intervention time may be 35 sessions, although the child would otherwise qualify for 40 sessions, including parental guidance.

o The child has had only a short period of exposure to the target language used in language therapy.

o Other circumstances based on the child’s individual situation and needs.

**Circumstances that may increase the recommended total intervention time**

The child has

o difficulties in several different developmental areas and thus also goals for intervention in several different areas.

o a current development phase of rapid progress.

o a sudden decrease of functional capacity related to the ability to interact, communicate, understand or use language, speak, eat, or swallow.

o secondary problems (e.g. behavioural and emotional problems) resulting from primary problems in language, literacy, communication, interaction, speech, eating or swallowing.

o considerable risk factors in their environment.

o more than two environments in which the people need consultative support.

o a therapy situation that requires extensive collaboration between the speech and language therapist and different stakeholders.

o significant delay in the onset of speech and language therapy.

o extensive problems with overall motor skills or sensory regulation related to difficulties in the ability to interact, communicate, understand or use language, speak, eat, or swallow.

o Other circumstances based on the child’s individual situation and needs.

**Circumstances that may increase the length of intervention sessions**

o The child or a family member uses augmentative or alternative communication.

o The sessions include a significant amount of consultative support.

o An interpreter is required for therapy sessions (the length of only those sessions is increased where an interpreter is present).

o The child's pace of functioning is slow and therefore more time than usual is required in activities.

o The child needs support in executive functioning skills or maintaining motivation.

o The child's sensorimotor regulation and preparation for activities requires additional time.

o The child has multiple therapy goals to work on simultaneously (e.g. eating and interaction).

o Therapy is carried out entirely within everyday situations.

o Other circumstances based on the child’s individual situation and needs.
